# Local Immunomodulatory Strategies to Prevent Allo‐Rejection in Transplantation of Insulin‐Producing Cells

**DOI:** 10.1002/advs.202003708

**Published:** 2021-07-14

**Authors:** Xi Wang, Natalie K. Brown, Bo Wang, Kaavian Shariati, Kai Wang, Stephanie Fuchs, Juan M. Melero‐Martin, Minglin Ma

**Affiliations:** ^1^ Department of Biological and Environmental Engineering Cornell University Ithaca NY 14853 USA; ^2^ Department of Cardiac Surgery Boston Children's Hospital Boston MA 02115 USA; ^3^ Department of Surgery Harvard Medical School Boston MA 02115 USA; ^4^ Harvard Stem Cell Institute Cambridge MA 02138 USA

**Keywords:** biomaterials, cell therapy, drug delivery, immunomodulation, type 1 diabetes

## Abstract

Islet transplantation has shown promise as a curative therapy for type 1 diabetes (T1D). However, the side effects of systemic immunosuppression and limited long‐term viability of engrafted islets, together with the scarcity of donor organs, highlight an urgent need for the development of new, improved, and safer cell‐replacement strategies. Induction of local immunotolerance to prevent allo‐rejection against islets and stem cell derived *β* cells has the potential to improve graft function and broaden the applicability of cellular therapy while minimizing adverse effects of systemic immunosuppression. In this mini review, recent developments in non‐encapsulation, local immunomodulatory approaches for T1D cell replacement therapies, including islet/*β* cell modification, immunomodulatory biomaterial platforms, and co‐transplantation of immunomodulatory cells are discussed. Key advantages and remaining challenges in translating such technologies to clinical settings are identified. Although many of the studies discussed are preliminary, the growing interest in the field has led to the exploration of new combinatorial strategies involving cellular engineering, immunotherapy, and novel biomaterials. Such interdisciplinary research will undoubtedly accelerate the development of therapies that can benefit the whole T1D population.

## Graphical abstract

An overview of different local immunomodulatory approaches in *β* cell replacement therapies is provided. Strategies in islet/*β* cell modification with immunomodulatory proteins immobilized on the cell surface or released to the local microenvironment, biomaterial platforms presenting or delivering immunomodulatory signals, and co‐transplantation of immunomodulatory cells are discussed.

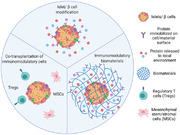

## 1 Introduction

### 1.1 Background of Type 1 Diabetes

Type 1 diabetes (T1D) is an autoimmune disease which affects millions of people worldwide and causes tremendous economic and societal burdens.^[^ ^1^ ^]^ T1D onset occurs when a patient's own immune system attacks pancreatic *β* cells, resulting in the deficiency of insulin, a hormone required for cells to metabolize glucose.^[^ ^2^ ^]^ Patients are typically diagnosed with fasting and/or postprandial hyperglycemia and islet‐associated autoantibodies.^[^ ^2^ ^]^ Daily infusions or injections of exogenous insulin are therefore required for the management of blood glucose concentrations; however, such treatments do not cure the disease or restore pancreatic function.^[^ ^3^ ^]^ Precise glucose balance, as controlled by islets, cannot be replicated with non‐physiologically responsive insulin delivery, even with the advancements in the development of long‐lasting insulin and insulin pumps.^[^ ^4^ ^]^ Also, insulin therapy can cause life‐threatening hypoglycemic unawareness in patients, which is a dangerous acute complication resulting from delayed insulin action.^[^ ^5^ ^]^ Moreover, long‐term glucose imbalance can lead to the development of hyperglycemia‐driven microvascular and macrovascular complications.^[^ ^3^ ^]^ Statistics show that patients with T1D have a much higher risk of cardiovascular disease than people without T1D, and about 30% of patients with T1D are diagnosed with chronic kidney disease.^[^ ^6^ ^]^ Last but not least, not only do patients with diabetes suffer from the physical discomfort of daily injections, which can generate scar tissue in the abdomen in the long term; but meal‐by‐meal disease management can also be a psychological burden to patients.

*β* cell replacement, on the other hand, offers the opportunity to achieve tighter physiological glycemic control and insulin independence.^[^ ^3^, ^7^ ^]^ In particular, the Edmonton Protocol—transplantation of human islets into the portal vein—has shown success among patients, many of whom sustained normoglycemia for three to 5 years after transplantation, with 10% of patients maintaining normoglycemia for more than 5 years.^[^ ^8^, ^9^, ^10^ ^]^ However, systemic administration of immunosuppressants is required to prevent allo‐rejection, which has adverse side effects, such as increased risk for infections and cancer, and affects the longevity of islet engraftment.^[^ ^11^, ^12^ ^]^ Also, once the suppressive therapy is terminated, resurgence or even elevation of autoimmune responses can occur. The idea of an encapsulation device, including microencapsulation and macroencapsulation, was raised decades ago as a method to deliver allogeneic islets while eliminating the need of immunosuppressive drugs.^[^ ^13^, ^14^, ^15^ ^]^ Once encapsulated, cells can be protected from host immune cells and in some cases antibodies, while still having access to nutrients, oxygen, and glucose. Tremendous work has been carried out in rodents to demonstrate the efficacy of using encapsulation devices to deliver foreign cells and reverse diabetes. Despite ongoing efforts to develop encapsulation devices to immuno‐protect or isolate allogeneic *β* cells, challenges remain for clinical application of this technology, including the acute loss of cells following implantation due to a lack of blood vessels and oxygen, the foreign body reaction against biomaterials and suboptimal survival of cells within the device due to inadequate mass transfer.^[^ ^13^, ^14^, ^15^ ^]^

**Figure 1:**
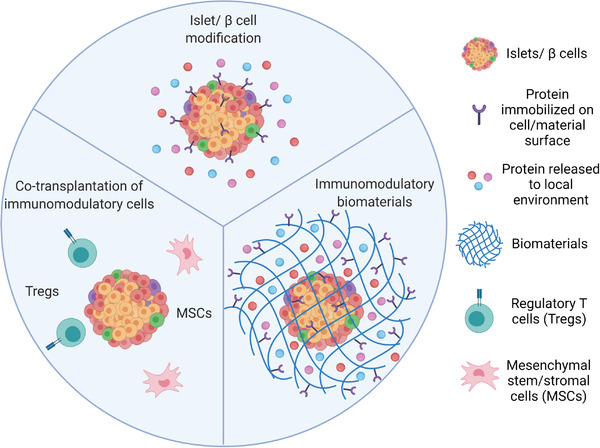
Approaches to achieve local immunomodulation in *β* cell replacement therapies including islet/*β* cell modification with immunomodulatory proteins immobilized on the islet/cell surface or released to the local microenvironment, biomaterial platforms presenting or delivering immunomodulatory signals or agents, and co‐transplantation of immunomodulatory cells such as regulatory T cells (Tregs) or mesenchymal stem/stromal cells (MSCs). (Only non‐encapsulation approaches or “open systems” will be reviewed in this paper.)

To address these limitations, research is being conducted to induce local immunotolerance, as an alternative approach to systemic immune suppression, to prevent allo‐rejection against islets, and stem cell derived *β* (SC‐*β*) cells without encapsulation.^[^ ^16^, ^17^, ^18^, ^19^, ^20^ ^]^ Local immunotolerance is defined as the lack of a destructive immune response against foreign cells or specific antigens in local areas while retaining the full capacity of the immune system to react to other foreign antigens.^[^ ^21^ ^]^ For advanced treatment of T1D with extensive *β* cell destruction, cell replacement therapy can, in principle, be applied in combination with local immunomodulatory or tolerogenic approaches to enable immunological acceptance of the graft. In this mini review, we discuss the immune responses to allografts, animal models used in T1D research, and different (nonencapsulation or “open system”) approaches to achieve local immunomodulation in *β* cell replacement therapies. We specifically discuss strategies in islet/*β* cell modification with immunomodulatory proteins immobilized on the islet/cell surface or released to the local microenvironment, biomaterial platforms presenting or delivering immunomodulatory signals or agents, and co‐transplantation of immunomodulatory cells such as regulatory T cells (Tregs) or mesenchymal stem/stromal cells (MSCs) (**Figure** **1**). The exciting preliminary research efforts in this domain portend that robust and long‐term restoration of normoglycemia using islet grafts will necessitate interdisciplinary and combinatorial therapy approaches.

### 1.2 Immune Responses during Allogeneic Islet Transplantation

**Figure 2:**
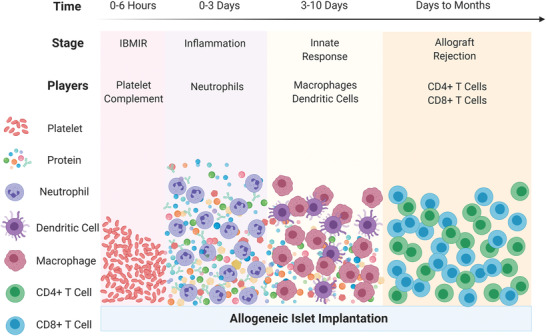
Temporal sequence of host immune responses to allogeneic islets, including instant blood mediated inflammatory reaction (IBMIR), inflammation, innate response, and allo‐rejection, the response time of which can range from hours to years following transplantation.

Similar to the immune response against pathogens, allogeneic islet‐elicited immune responses may be divided into four stages, including the instant blood mediated inflammatory reaction (IBMIR), inflammation, innate response, and allo‐rejection (**Figure** **2**), the response time of which can range from hours to years following transplantation.^[^ ^16^, ^22^ ^]^

In the first stage of the allogeneic immune response, transplantation‐induced tissue injury results in local inflammation. Depending on the transplantation route, different inflammatory mechanisms may be involved in this process. For example, in intrahepatic islet transplantations, an IBMIR—triggered by islet contact with hepatic vasculature blood—boosts proinflammatory cytokine release, including interleukin‐6 (IL‐6), interleukin‐8 (IL‐8), interferon gamma‐induced protein 10 (IP‐10), and monocyte chemoattractant protein‐1 (MCP‐1).^[^ ^23^, ^24^ ^]^ Activation of complement proteins C5a and C3a, followed by the complement cascade, also contributes to islet inflammation and death.^[^ ^25^ ^]^ These complement proteins further upregulate adhesion molecules on the endothelial cell surface, thereby recruiting more leukocytes from the blood, which in turn augments inflammation. These factors rapidly recruit innate immune cells, such as macrophages and neutrophils, to the transplantation site and cause further islet inflammation and death.^[^ ^26^, ^27^ ^]^ On the other hand, islet transplantation in extrahepatic sites has substantially reduced the IBMIR. However, transplantation by itself as a tissue insult, given the processes of islet procurement, ischemia, and reperfusion injury, releases proinflammatory or “danger” signals.^[^ ^28^, ^29^ ^]^ These signals also induce local inflammation, characterized by enhanced cytokine production, metabolic stress, and influx and activation of innate immune cells. As such, independent of allogeneic islet transplantation route performed, alloantigen‐independent and islet localized inflammation are inevitable first steps of the host response against the graft.

Following local inflammation, recipient dendritic cells (DCs) are recruited to the transplanted islets by inflammatory and “danger” signals.^[^ ^30^ ^]^ They phagocytose damaged cells in allogeneic islets and subsequently present alloantigens on their surfaces. As professional antigen presenting cells (APCs), DCs express both major histocompatibility complex (MHC) class I and II, thus enabling the priming of both CD8^+^ cytotoxic and CD4^+^ helper T cells.^[^ ^31^ ^]^ These inflammatory and “danger” signals also function as coactivating signals to mature DCs. After maturation, the alloantigen presenting DCs migrate to lymph nodes that drain transplanted islets, and to other secondary lymphoid tissues.^[^ ^31^ ^]^ Collectively, maturation of alloantigen‐loaded DCs bridges the acute, innate, and chronic adaptive immune responses against allogeneic islets.

**Figure 3:**
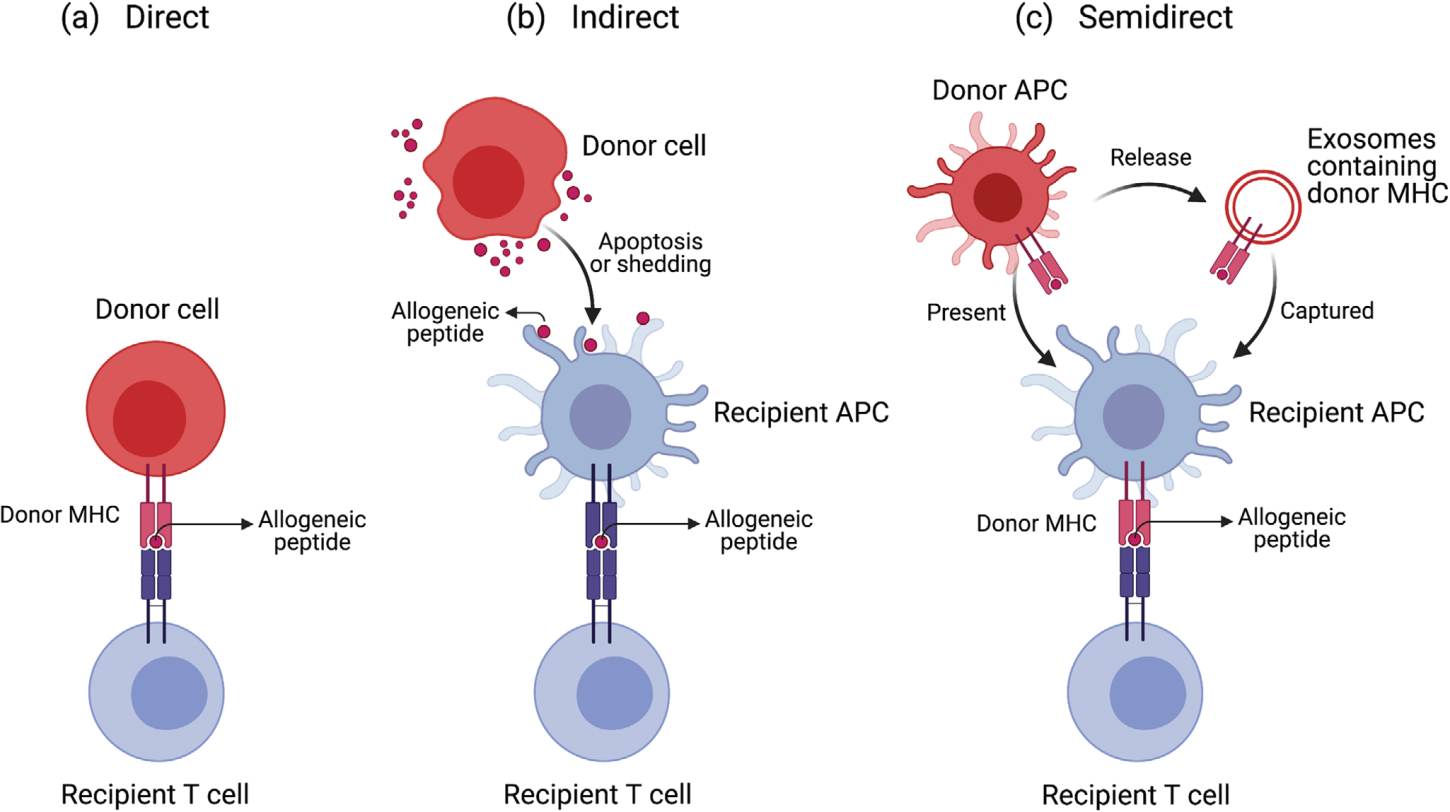
Three mechanisms of allorecognition: a) direct, b) indirect, and c) semidirect recognition. Reproduced with permission.^[34]^ Copyright 2020, Springer Nnature.

In secondary lymphoid tissues, mature DCs present alloantigens to activate recipient T cells and expand those that are alloantigen‐specific.^[^ ^31^ ^]^ T cell allorecognition occurs in three different ways (e.g., direct, semidirect, and indirect recognitions) (**Figure** **3**).^[^ ^32^, ^33^, ^34^ ^]^ The major distinction between the various allorecognition pathways is whether the MHC itself or the peptide it presents leads to T cell receptor (TCR) activation. In direct allorecognition, donor‐specific and allogeneic MHC induces the activation of recipient T cell. These allogeneic cells can be APCs or any allogeneic MHC expressing cells from the donor. Similarly, in semidirect allorecognition, the allogeneic MHC activates the T cell. However, unlike in direct allorecognition, these allogeneic MHC molecules are now presented by recipient DCs and other APCs, which are acquired through phagocytosis of allogeneic cells. Therefore, in these two recognition modes, peptide origin is irrelevant. On the other hand, in indirect allorecognition, allogeneic proteins are degraded by recipient APCs, and the derived allogeneic peptides are presented by autologous MHC. These allogeneic peptide‐autologous MHC molecules are recognized by TCR. Thus, in this recognition mode, peptide origin is the key for T cell activation. Though all three allorecognition modes are possible in secondary lymphoid tissues, a recent study by Hughes et al., suggests that direct allorecognition is more prevalent in islet transplantation when the activated T cell migrate to the graft.^[^ ^35^ ^]^ Besides TCR engagement with peptide‐MHC molecules, a costimulatory signal, commonly provided by CD80 or CD86 ligands on APCs that interact with CD28 on T cells, is also necessary for T cell activation. Alternatively, CD40 on activated APCs can interact with CD40L (CD154) on T cells to fully activate them.^[^ ^36^ ^]^ In summary, activation of alloantigen‐specific T cells by DCs and other APCs marks the beginning of adaptive immune responses.

Finally, migration of the activated T cells to the graft causes more damage to and eventually destroys the transplanted islet.^[^ ^37^ ^]^ In this process, CD8^+^ T cells directly destroy islet cells by secreting cytotoxic molecules such as perforin and granzyme B, while CD4^+^ T cells indirectly contribute by boosting CD8^+^ T cells and secreting more inflammatory cytokines, such as interferon gamma (IFN*γ*) and tumor necrosis factor alpha (TNF*α*). These cytokines recruit more immune cells to reject islets. Though T cells are historically the primary effectors in immune responses against allogeneic islets, there is increasing evidence that B cells are paramount in long‐lasting chronic rejection of allogeneic transplants.^[^ ^38^, ^39^, ^40^ ^]^ For example, by phenotyping alloreactive T cell subsets in B cell deficient mice and comparing differentiation of activated alloreactive T cells adoptively transferred into naïve mice with or without B cells, Ng et al., demonstrated that B cells contributed to alloreactive T cell differentiation into memory T cell.^[^ ^39^ ^]^ They further found that antibody production was dispensable for B cells to mediate chronic allograft rejection.^[^ ^40^ ^]^ These studies indicate that besides alloantibody production, the role of B cells in long‐lasting allo‐rejection may include alloantigen presentation and shuttling, maintenance of lymphoid architecture that is important for immune responses, formation of immune complexes that amplifies the interactions between DCs and T cells, and supply of costimulatory signals and secretion of cytokines to facilitate T cell differentiation.^[^ ^41^, ^42^, ^43^, ^44^, ^45^, ^46^ ^]^ Given the complexity of allogeneic responses, new animal models need to be developed to further understand the function of B cells in allo‐rejection.

Besides allo‐rejection, islet allografts may also suffer from preexisting autoimmunity against the islets in the recipient. This recurrence of islet autoimmunity may further accelerate the destruction of transplanted islets. A significant evidence that reinvigoration of islet autoimmunity contributes to recurrence of T1D comes from the detection of autoantibodies and autoreactive CD4^+^ T cells against glutamic acid decarboxylase (GAD), tyrosine‐like phosphatase protein IA‐2, islet‐specific glucose‐6‐phosphatase catalytic subunit‐related protein (IGRP), and cation efflux transporter ZnT8 in patients that developed hyperglycemia again after receiving pancreas transplants.^[^ ^47^, ^48^, ^49^ ^]^ Unlike allo‐rejection that requires T cell priming by alloantigen‐presenting APCs, these pathogenic autoimmune B and T cells can be rapidly reactivated upon confrontation with the above autoantigen. The de facto “boosting” of these pathogenic cells makes them clonally expand and secrete effector autoantibodies and inflammatory cytokines, which further amplify the autoimmune reaction. However, whether allo‐rejection or autoimmunity contributes more to allograft destruction is still unclear. Two studies have investigated the role of autoimmunity in islet allograft rejection.^[^ ^50^, ^51^ ^]^ Makhlouf et al., compared the survival of islet transplants with cardiac transplants, the former subject to autoimmunity while the latter not, and found that in the presence of CD80/86 and CD40L blockade, MHC matching favored heart but not islet survival, while the absence of full or MHC class II matching resulted in no difference between the survival of the heart and the islet.^[^ ^50^ ^]^ This indicates that autoimmunity played a significant role in islet allo‐rejection. On the other hand, making use of islet‐specific BDC‐2.5 TCR transgenic CD4^+^ autoimmune T cells, Kupfer et al., demonstrated that these pathogenic T cells can also destroy islet allografts that are MHC class II mismatched.^[^ ^51^ ^]^ This again suggests that the destruction of islet allografts is a complicated process that involves both allo‐rejection and recurrence of autoimmunity. More studies are needed to deconvolute each one's contribution.

Though numerous types of immune cells are involved, the multi‐stage and orchestrated immune responses against allogeneic islet transplant provide manifold checkpoints for rejection mitigation. Some of the mechanisms discussed above have been successfully exploited to develop allogeneic islet protection strategies, which will be reviewed in the following sections.

### 1.3 Animal Models for T1D

In studying T1D and testing local immune tolerance therapies, it is important to consider the selection of suitable in vitro and in vivo models. A lack of ideal and translational cell or animal models has presented challenges for these developments; however, there are still a multitude of viable options that can recapitulate different aspects of disease autoimmunity and progression.

Single‐antigen transgenic mouse models have been created to mimic autoimmunity by adoptive transferring diabetogenic cells, such as *β* cell specific CD4^+^ and CD8^+^ T cell clones from BDC2.5 and NOD8.3 mice, into NOD‐SCID mice.^[^ ^16^, ^52^, ^53^, ^54^ ^]^ These models are simple because of their single‐antigen characteristic and are valuable for studying disease initiation. Additionally, pathogen‐induced mouse models, such as those infected using lymphocytic choriomeningitis virus (LCMV), can be helpful in studying T cell autoimmune activity in a rapid yet non‐spontaneous T1D onset.^[^ ^53^ ^]^ However, pathogen‐induced models fail to compensate for epitope spreading.^[^ ^16^ ^]^ Chemical injections, such as streptozotocin (STZ), provide an opportunity for time‐ and severity‐controlled induction of *β* cell ablation without an accompaniment of autoimmune mechanisms.^[^ ^55^ ^]^ Chemical induction methods are therefore typically used to study rejection of allogeneic *β* cell transplant in the absence of complex autoimmune responses. Humanized mouse models provide an additional option for T1D immunotherapy study.^[^ ^56^ ^]^ However, they lack essential homology required for full effector function.

The nonobese diabetic (NOD) mouse is known as the “gold standard” as a model of T1D due to its similarities to human T1D, and develops insulitis via leukocytic aggregation and lymphocytic infiltration, which progresses spontaneously into diabetes.^[^ ^52^, ^53^, ^57^ ^]^ However, important differences in NOD diabetic onset include a blatant sex bias, with a penetrance of 70–90% for females yet only 10–40% in males, which does not hold true in human T1D.^[^ ^52^ ^]^ Also, it should be noted that NOD mice have been inbred in laboratories for many years. Many genes and phenotypes have been enriched, but not all of them are related to T1D.^[^ ^57^ ^]^ It has also been reported that some drugs fail to translate from NOD mice to human.^[^ ^58^ ^]^ Within the realm of rodent models, both BB (BioBreeding) and LEW.1AR1/‐iddm rats develop spontaneous diabetes. BB rats have been specifically noted as ideal for investigating tolerance induction in islet transplantation.^[^ ^59^ ^]^ However, following the development of insulitis, BB rats are lymphopenic, with significantly decreased counts of CD4^+^ and CD8^+^ T cells, which does not align with human T1D presentation.^[^ ^59^ ^]^ LEW.1AR1/‐iddm rats become diabetic with a roughly 60% success rate and differ from both NOD mice and BB rats in that they do not manifest other autoimmune conditions.^[^ ^60^ ^]^ Though spontaneous diabetes has been reported in large animals such as cats, dogs, and pigs, it is relatively rare and unpredictable in onset.^[^ ^61^ ^]^ Few studies have been carried out in outbred large animal models, partially due to the fact that large animals have longer life cycles and higher maintenance costs than inbred rodents.^[^ ^55^ ^]^

Exciting advances in cell culture techniques have recently brought about the creation of an in vitro model of T1D utilizing SC‐*β* cells, which has potential to study T1D disease progression in vitro.^[^ ^62^, ^63^ ^]^ In summary, there is a great variety of models currently in place for the study of T1D, and each has its own strengths and limitations. It is essential to consider testing new techniques in more than one model system, to allow for the consideration of different aspects of disease presentation and its signaling pathways, all in the interest of optimal translational potential. Though challenges exist in studying T1D in vitro and in vivo, there are progresses in strategies inducing immune tolerogenic microenvironment to assist islet transplantation.

## 2 Local Immunomodulation Strategies

### 2.1 Islet/Beta Cell Modification

#### 2.1.1 Modification of Islets

**Figure 4:**
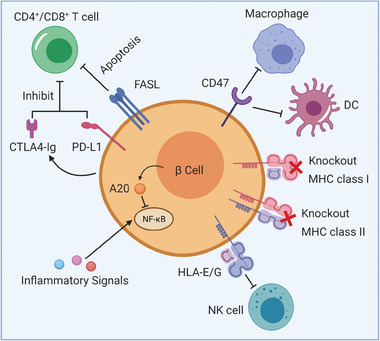
Engineering *β* cells to evade immune responses through knockout of MHC class I and MHC class II, and over‐expression of immune‐modulatory factors including PD‐L1, CTLA4, Fas‐ligand, human leukocyte antigen‐G (HLA‐G), CD47, and A20.

In the pursuit of engineering islet cells capable of evading an immune attack, different immune cloaking strategies have been developed. For example, Iwata et al., reasoned that since most transplanted islets are destroyed by the IBMIR, immobilizing anticoagulant enzymes including urokinase (UK) and thrombomodulin (TM) onto the surface of islets could reduce thrombus formation.^[^ ^64^, ^65^, ^66^ ^]^ After modification, islet morphologies, volumes, and function in vitro were not affected, however, the function of the implanted islets in intrahepatic sites was unexplored. Another surface modification strategy includes biotinylating the islet membrane to conjugate streptavidin‐Fas‐ligand (SA‐FasL) proteins, which is known to activate the programmed death of T cells when engaged with Fas (CD95).^[^ ^67^ ^]^ (**Figure** **4**). After transplanting SA‐FasL engineered islets into diabetic allogeneic hosts in conjunction with a short‐term course (14 days post‐transplantation) of rapamycin treatment, robust localized tolerance was induced by CD4^+^CD25^+^Foxp3^+^ regulatory T (Treg) cells and the reversal of diabetes was noted.^[^ ^68^, ^69^ ^]^

Beyond the Fas ligand, other immune checkpoints have also been applied in the modification of *β* cells. Considering the pivotal role of CD47 as a negative checkpoint for myeloid cells, streptavidin‐CD47‐engineered islets showed a mitigated IBMIR, and enhanced islet engraftment compared to controls.^[^ ^70^ ^]^ In addition to surface modification, ex vivo gene therapy strategies using adenovirus or adeno‐associated virus (AAV) were explored to label islet cells with immunomodulatory proteins including A20 and programmed death‐ligand 1 (PD‐L1)/cytotoxic T‐lymphocyte‐associated protein 4 (CTLA4) (Figure 4). Grey et al., discovered that A20 inhibited cytokine‐induced apoptosis by suppressing nuclear factor‐*κ*B (NF‐*κ*B) dependent gene activation in islets. Overexpression of A20 by means of adenovirus‐mediated gene transfer protected islets from TNF*α*, interleukin 1 beta (IL‐1*β*) and IFN*γ*‐induced apoptosis.^[^ ^71^ ^]^ They further showed that increasing A20 expression in allogeneic islet grafts lead to long‐term survival for about 45% of recipients, and more than 80% survival when combined with rapamycin injection. Mechanistically, overexpression of A20 raises inflammatory thresholds to favor immune tolerance mediated by Treg cells.^[^ ^72^ ^]^ Ikeda et al., focused on enhancing immune checkpoints via forced expression of PDL1‐CTLA4Ig in murine islets through the delivery of *β*‐cell targeted AAV. Implantation of PDL1‐CTLA4Ig‐expressing MHC‐matched islets into diabetic NOD mice resulted in protection of allogeneic islets from acute rejection, although islet grafts were eventually rejected.^[^ ^73^ ^]^ Freddy et al., developed Ins2‐CCL21 transgenic NOD mice which express chemokine (C‐C motif) ligand 21 (CCL21) in pancreatic *β*‐cells and avoided developing autoimmune diabetes.^[^ ^74^ ^]^ All the above studies represent localized immunosuppression strategies which allow for reduced or even no systemic immunosuppression, thus decreasing opportunistic infections.

#### 2.1.2 Modification of Stem Cell Derived *β* Cells

Similar to other organ transplantations, the widespread application of islet transplantations is limited by donor shortage. As such, significant progress has been achieved in the generation of SC‐*β* cells with close resemblance to their in vivo counterparts regarding metabolic control.^[^ ^75^ ^]^ However, the prevention of immune detection and destruction of such cells by the host immune system remain elusive.^[^ ^76^ ^]^ To achieve immune cloaking in human embryonic stem cells (hESCs) and their progenies, genome editing tools, most notably the CRISPR/Cas9 system, have been applied to disrupt the MHC components and over‐express immune‐modulatory factors including PD‐L1, CTLA4, human leukocyte antigen‐G (HLA‐G), and CD47 (Figure 4).^[^ ^77^, ^78^, ^79^, ^80^ ^]^ Beta 2 microglobulin (*β*2M) facilitates the proper folding and expression of MHC class I proteins on the cell surface while the MHC Class II Transactivator (CIITA) binds to sequence elements in the MHC class II gene promoter. Although targeted disruption of the *β*2M and CIITA generates modified hESCs which significantly decreases the alloimmune response, complete ablation of MHC molecules renders the hESCs vulnerable to natural killer (NK) cells‐mediated killing. To overcome this, overexpression of HLA‐G or CD47 was found to enable hESCs to evade NK‐cell‐mediated lysis.^[^ ^78^, ^79^ ^]^ Interestingly, HLA‐C‐retained induced pluripotent stem cells (iPSCs) while disrupting both HLA‐A and HLA‐B alleles could evade T cells and NK cells in vitro and in vivo.^[^ ^77^ ^]^ To further validate the immunogenicity of the modified stem cells, human stem cells, and their derivatives, including endothelial cells and cardiomyocytes, were implanted in allogeneic humanized mice to test the immune response. Compared to wild type stem cells, modified stem cells could form teratoma for more than 50 days while endothelial cells could survive for more than 30 days without immunosuppression.^[^ ^79^ ^]^ The allogeneic immune responses toward SC‐*β* cells may be overcome through the application of autologous SC‐*β* cells, however, recurrent autoimmunity following transplantation is still expected to destroy the *β* cells.^[^ ^62^ ^]^ By performing an unbiased genome‐wide screen in NOD mice, deleting a gene called *RNLS* rendered the mouse *β* cells and SC‐*β* cells resistant to autoimmune killing through diminishing immune recognition and conferring endoplasmic reticulum (ER) stress resistance.^[^ ^81^ ^]^ Overexpression of PD‐L1 even protected SC‐*β* cells xenografts in immune‐competent mice for 50 days while restoring the glucose homeostasis.^[^ ^82^ ^]^ Taken together, the genetic modification of stem cells holds promise for creating hypoimmunogenic universal cell grafts which are invisible to the immune system and eliminate the need for immunosuppressive drugs.

However, current studies are still preliminary, and more work is needed to achieve the ultimate goal of treating T1D patients with modified SC‐*β* cells without immunosuppression. While testing allogeneic human cells in humanized mice provides invaluable information, the models cannot fully recapitulate the human immune response and conclusions drawn from these studies may be limited. In addition, hypoimmunogenic modifications of stem cells can lead to escape from the immune surveillance which is key to prevent infection and tumor formation. Lastly, more efficient modifications with less off‐target effects warrant further explorations and investigations.

### 2.2 Biomaterial Strategies

**Figure 5:**
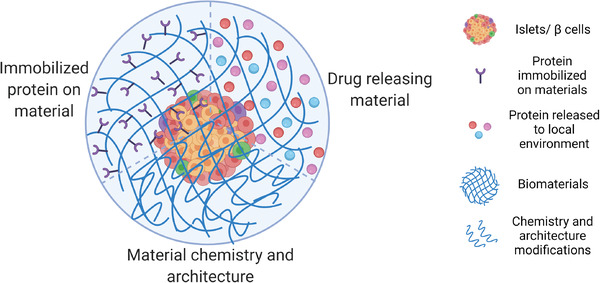
Biomaterial strategies to modulate immune response including biomaterials immobilized with immunomodulatory factors, those with controlled release of immunomodulatory agents and those with special material chemistry and architecture.

Through adding epitopes directly onto the surface of biomaterials, loading biomaterial scaffolds and particles with immunomodulatory factors, and modifying the biomaterial itself, local immunomodulation can enhance graft acceptance by allowing for controlled presentation, loading, and release of compounds, ultimately creating a more favorable microenvironment (**Figure** **5**). In this section, we review a variety of findings that have either been established as proof of concept, undergone testing in diabetic animal models, or have found success in related autoimmune diseases and may be applied to the field of islet transplantation.

#### 2.2.1 Immobilizing Proteins on Biomaterial Surface

Modifying the surface of biomaterials used in graft transplantation offers an attractive alternative to systemic tolerance induction. A variety of factors can be attached to biomaterial surfaces, perhaps even in combination to modulate multiple components of the immune response at once (**Table** **1**). Immunomodulatory microgels displaying streptavidin coupled with Fas ligand (FasL) significantly extended islet allograft survival.^[^ ^83^ ^]^ When combined with acute rapamycin treatment, grafts were prolonged to an excess of 200 days and restored normoglycemia in mice models.^[^ ^83^ ^]^ This same approach was applied to poly (lactide‐*co*‐glycolide) (PLG) scaffolds, and seeding islets into these allogeneic mice, with temporary rapamycin administration, led to a comparable normoglycemia and survival outcome.^[^ ^84^ ^]^ More recently, it was reported that biotinylated poly (ethylene glycol) (PEG) microgels conjugated with streptavidin/PD‐L1 (SA‐PD‐L1) extended the survival of allogeneic islets up to 2 months. Under the short‐term rapamycin administration, recipients receiving PD‐L1 modified microgels exhibited long‐term (>100 days) function. They reasoned that PD‐L1 presenting microgels promoted a tolerogenic microenvironment by increasing the CD4 Treg population.^[^ ^85^ ^]^ Though not yet explicitly applied to diabetes therapeutics, functionalized ligands have been shown to locally enhance immune cell populations and provide an avenue for targeted delivery. Coating tacrolimus‐loaded nanoparticles with MECA79 antibody, which targeted them to peripheral lymph nodes, enhanced survival of murine heart allografts from 7 days to a median survival of 11 days.^[^ ^86^ ^]^

**Table 1:** Examples of protein immobilization and drug delivery for local immunomodulation

Biomaterial approach	Material and/or islet‐containing construct	Modification (drug, antigen)	Model	Notable immunomodulatory effects	Reference
Immobilized protein on material	Microgels	FasL modified microgels	Diabetic mice, rapamycin treatment	Significantly extended allograft survival to 200+ days, restored normoglycemia	^[^ ^83^ ^]^
	PLG scaffold conjugated with biotin	FasL modified scaffold	Diabetic mice, rapamycin treatment	Significantly extended allograft survival to 200+ days, restored normoglycemia	^[^ ^84^ ^]^
	PEG microgels	PD‐L1 modified microgels	Diabetic mice, rapamycin treatment	Significantly extended allograft survival to 100+ days, restored normoglycemia	^[^ ^85^ ^]^
Drug‐releasing material	PLG scaffold	Added TGF‐*β*1	Diabetic mice	Decreased production of proinflammatory cytokines; dose‐dependent decrease in leukocyte infiltration, and delayed allograft rejection	^[^ ^97^ ^]^
	PDMS scaffold	Scaffold loaded with dexamethasone	Diabetic mice	Promoted M2 macrophage polarization. Lower percentages of dexamethasone restored stable normoglycemia post‐transplant more effectively	^[^ ^101^ ^]^
	MicroPLGA micelles	Dexamethasone and CTLA4‐Ig	MHC‐mismatch mice	Decreased proinflammatory cytokines within grafts and improved glucose tolerance. Restored insulin independence for 60 days	^[^ ^103^ ^]^
	Biohybrid device: osmotic pump with central sprinkler combined with PLA microspheres	Pump contained dexamethasone phosphate, Microspheres loaded with LE	Diabetic rats	Delayed islet graft rejection for 5–6 weeks	^[^ ^104^ ^]^
	PLG scaffolds	IL‐33 Released	Diabetic mice	Enriched Treg counts at graft site. Rejection delayed with median graft survival of 33 days, but slowed engraftment time	^[^ ^124^ ^]^
	Human acellular dermal matrix scaffolds	Fc fusion protein and CTLA4‐Ig bioprinted onto scaffolds	MHC‐mismatch mice	Expansion of Treg cells, favorable cytokine expression in microenvironment, enhanced graft acceptance with 71‐day survival time, normoglycemia for >28 days	^[^^125^, ^126^^]^
	PDMS scaffolds	Local FTY‐720 delivery	Diabetic mice	Not successful in promoting allograft acceptance, detrimental to islets	^[^ ^127^ ^]^
	PHBV and PCL nanofibers	Loaded with FTY‐720	Diabetic mice	Islets transplanted following 2 weeks of pretransplanting with nanofibers, normoglycemia observed	^[^ ^128^ ^]^
	Injectable matrigel	Clodrosome delivery	Diabetic mice	Depleted macrophage populations, lowered IL‐1*β* and TNFɑ concentrations, median transplanted islet survival >60 days	^[^^132^, ^133^^]^
	APA microspheres made of alginate with PLL coating	Anti‐CD3*ε* or anti‐CD95 monoclonal antibodies	Diabetic mice	Significantly dampened autoimmune response, islet‐protecting effect	^[^ ^134^ ^]^
	Matrigel containing PLGA microparticles	FK506 encapsulated in microparticles	Diabetic mice	Impeded T‐cell activation, euglycemia and improved islet survival	^[^ ^135^ ^]^

#### 2.2.2 Drug‐Releasing Biomaterials

The enhancement of biomaterials to deliver immunomodulatory molecules through the use of nanoparticles, microparticles, and scaffolds has been well‐studied.^[^ ^87^, ^88^, ^89^, ^90^ ^]^ Many of these approaches are designed with disease treatment in mind, and so work to prevent or delay the onset of T1D and have been extensively reviewed elsewhere.^[^ ^16^ ^]^ Local release of therapeutics is highly favored over systemic administration, as many of these compounds can have harmful or adverse immune‐dampening effects when left to broadly circulate. Confining drug release to the graft site encourages a more targeted course of action, given that there is likely a higher concentration of the desired substance in the transplant microenvironment. Loading multiple factors into biomaterial fabrications has the potential to amplify a desired effect or target multiple facets of the immune system simultaneously.^[^ ^91^ ^]^ This approach has been explored by multiple groups and will be briefly reviewed in the following (Table 1).

##### Anti‐Inflammatory Compounds

The use of biomaterials to sustain targeted delivery of anti‐inflammatory compounds and mediate graft rejection has shown promise in a number of studies. The local delivery of transforming growth factor‐*β*1 (TGF‐*β*1) is desirable as systemic administration of the cytokine can have adverse effects on graft fibrosis.^[^ ^92^ ^]^ It also has a significant and versatile role within the immune system, contributing to anti‐inflammatory responses, the conversion of CD4^+^ T cells into Tregs, Treg development and survival, and macrophage, NK cell, and neutrophil activity.^[^ ^93^, ^94^, ^95^, ^96^ ^]^ When loaded within an islet‐containing microporous PLG scaffold transplanted into the epididymal fat pad, TGF‐*β*1 release aided in mitigating the immune response toward allogeneic islets.^[^ ^97^ ^]^ Proinflammatory cytokines including TNF*ɑ*, interleukin 2 (IL‐2), and MCP‐1 decreased in production, and the degree of leukocyte infiltration decreased in a dose‐dependent manner with respect to the concentration of TGF‐*β*1 loaded within the scaffold. Fending off graft rejection allowed for the restoration of normoglycemia, though this effect did not exceed 28 days after islet transplantation.^[^ ^97^ ^]^

Dexamethasone is an attractive candidate given its ability to promote the conversion of macrophages into their more favorable anti‐inflammatory M2 phenotype.^[^ ^16^, ^98^, ^99^, ^100^ ^]^ Like many immunomodulatory options, there is a balance to be maintained, as excessive administration can impair the health and glucose responsiveness of grafts, and even cause death of insulin‐secreting cells.^[^ ^101^, ^102^ ^]^ In one study, various concentrations of dexamethasone loaded into polydimethylsiloxane (PDMS) scaffolds were transplanted with islets into diabetic mice and analyzed for graft efficacy and glycemic impact.^[^ ^101^ ^]^ The authors found that lower percentages of dexamethasone, namely 0.25% and 0.1% w/w, restored stable normoglycemia for about 2 weeks to 100% and 90% of recipients, respectively.^[^ ^101^ ^]^ The pairing of dexamethasone with T‐cell‐associated CD28 antagonist CTLA4‐Ig in micelles comprised of micro‐poly (lactic‐*co*‐glycolic acid) (PLGA) led to enhanced immune acceptance in a murine islet allograft model. The micelle delivery restored insulin independence for 60 days in 80% of recipients. Notably, the addition of dexamethasone to the CTLA4‐Ig micelles resulted in a reduced presence of proinflammatory cytokines such as IL‐1*β*, interleukin‐10 (IL‐10), and IFN*γ* within grafts, as well as improved glucose tolerance.^[^ ^103^ ^]^ A preliminary study combining two glucocorticoids—dexamethasone phosphate in an osmotic pump with a central sprinkler, and poly (lactic acid) (PLA) microspheres containing loteprednol etabonate (LE)—as part of a biohybrid device was also able to delay graft rejection, though not without further stability issues to be addressed.^[^ ^104^, ^105^ ^]^ The conjugation of dexamethasone to a peptide nanofiber gel has also been proposed as a local delivery system.^[^ ^106^ ^]^

Curcumin is a plant‐based anti‐inflammatory compound that can be incorporated into drug‐releasing biomaterial to create a local immunosuppressive environment. Curcumin has been popularized as an oral supplement; however, it is important to note that the bioavailability of curcumin is very low, and it is difficult to build up high circulating concentrations of curcumin in the body due to the speed with which it is metabolized.^[^ ^107^ ^]^ There have also been reports of curcumin inducing DNA damage on mammalian cells, so the incorporation of this compound into potential biomaterial approaches would require careful consideration of dosage with regards to the local microenvironment.^[^ ^108^, ^109^, ^110^, ^111^ ^]^ More positively, it has been previously demonstrated that curcumin's antioxidant properties can have protective effects on islet cells against cytokine damage.^[^ ^112^ ^]^ Treatment with curcumin has also had generally promising outcomes related to islets in a myriad of studies.^[^ ^113^, ^114^, ^115^ ^]^Though not yet tested in a mouse allograft model, the preparation of islet hetero‐spheroids embedded with curcumin‐loaded PLGA microspheres showed benefits in both cell viability and apoptosis mitigation.^[^ ^116^ ^]^ Delivery of curcumin‐loaded R3V6 amphiphilic peptide micelles to INS‐1 cells had a similar anti‐apoptotic outcome in hypoxic conditions.^[^ ^117^ ^]^ Curcumin has also reduced fibrosis when co‐encapsulated with islets,^[^ ^118^ ^]^ and has been successfully incorporated into various other biomaterial systems as well as exosomes as a means of nanoparticle delivery.^[^ ^119^, ^120^, ^121^, ^122^, ^123^ ^]^

It is additionally worth mentioning that a host of related anti‐inflammatory compounds often implicated in clinical islet transplantation such as anti‐TNF and interleukin‐1 receptor antagonist (IL‐1Ra) have been effectively incorporated into biomaterial platforms, but not yet demonstrated in graft‐based systems and are reviewed elsewhere.^[^ ^16^ ^]^ Regardless, incorporating anti‐inflammatory agents into biomaterials is an interesting approach to promote islet allograft acceptance.

##### Immunomodulatory Agents

The encouragement of allograft tolerance through immunomodulatory biomaterials extends to the release of immunomodulatory drugs and factors which serve to upregulate protective components of the immune system such as Treg cells. Releasing an M2‐polarizing cytokine, interleukin‐33 (IL‐33), from PLG scaffolds housing allogeneic islets had a mixed effect on overall acceptance of a transplant in diabetic mice—rejection was mitigated for 19 days, but the engraftment process proved difficult with some early reversible dysfunction.^[^ ^124^ ^]^ Importantly, the graft microenvironment itself was changed to help promote islet survival. IL‐33 presence in the allogeneic model enriched counts of beneficial Treg cells, shifting their percentage in the population of total CD4^+^ cells from 40% to 75%, while also decreasing graft‐rejecting CD8^+^ cells.^[^ ^124^ ^]^ Previously described CTLA4‐Ig functions to block CD28‐mediated T‐cell activation, and the bioprinting of this immunoglobulin/Fc fusion protein onto human acellular dermal matrix scaffolds permitted its local delivery.^[^ ^125^, ^126^ ^]^ Co‐transplantation of allogeneic islets in this matrix resulted in enhanced graft acceptance and normoglycemia in mice, accompanied by Treg expansion and a favorable shift in cytokine expression profiles.

Reagents that work to keep activated T cells within lymph nodes have also been explored for islet graft acceptance. Fingolimod hydrochloride, FTY‐720, an immune factor capable of modulating T‐cell migration, was integrated into PDMS scaffolds for islet co‐transplantation, though local delivery of FTY‐720 did not see as beneficial of results for allograft acceptance and correction of diabetes as systemic administration has shown previously. Fingolimod doses higher than 0.5% w/w negatively impacted engraftment of the cells, whereas lower concentrations did not have the desired immunoregulatory effect.^[^ ^127^ ^]^ Bowers et al., showed that FTY‐720‐loaded poly (3‐hydroxybutyrate‐*co*‐3‐hydroxyvalerate) (PHBV) and poly (*ε*‐caprolactone) (PCL) nanofibers could still be used if preimplanted and given an adjustment period of at least 2 weeks to adapt to the new environment before islets were added.^[^ ^128^ ^]^

The development of biomaterials to expand Treg populations as well as eliminate antigen‐specific T cells and manipulate the activation of APCs has shown promise for a host of autoimmune diseases. We point to several thorough reviews on the local as well as systemic scale for immunomodulatory material engineering and microparticle‐ and nanoparticle‐based autoimmune vaccine development.^[^ ^87^, ^88^, ^89^, ^129^, ^130^, ^131^ ^]^

##### Immunosuppressive Agents

Lastly, biomaterials can be coupled with drugs and compounds that induce tolerance by actively downregulating immune activation, such as by inhibiting APC responses, dampening cytokine signaling, and suppressing T‐cell recruitment. Injectable hydrogel Matrigel was used to aid in the co‐delivery of liposomal clodronate (also known as Clodrosome), an agent known to cause depletion of macrophages, with islets.^[^ ^132^, ^133^ ^]^ Indeed, macrophage inhibition had the desired effect of reversing diabetes in mice receiving islet, Matrigel, and Clodrosome packaged together, with transplanted islets surviving for more than 60 days. Effectiveness was confirmed by analyzing levels of cytokines TNFɑ and IL‐1*β* in both serum and the Matrigel themselves. The presence of Clodrosome lowered concentration of IL‐1*β* and TNFɑ in the islet‐containing Matrigel. A similarly interesting study demonstrated that antibodies can be locally released from microspheres consisting of alginate coated with poly‐l‐lysine (PLL) and containing a further outer layer of more alginate (alginate‐PLL‐alginate, APA). APA spheres loaded with either anti‐CD3*ε* or anti‐CD95 monoclonal antibodies were implanted along with an islet scaffold into diabetic mice, and autoimmune responses were significantly dampened—even to the point of mice being able to tolerate a typically lethal concentration of anti‐CD95.^[^ ^134^ ^]^ The co‐delivery of FK506—alternatively referred to tacrolimus, and often used in clinical islet transplantation—encapsulated within PLGA microparticles as part of a Matrigel containing islets impeded T‐cell activation to provide both an advantageous transplant environment and euglycemia to diabetic mice. The subcutaneously injected Matrigel may also have dual action as an immuno‐protective barrier.^[^ ^135^ ^]^ Adding immunomodulatory aryl hydrocarbon receptor (AhR) ligand 2‐(1′H‐indole‐3′‐carbonyl)‐thiazole‐4‐carboxylic acid methyl ester (ITE) and T1D autoantigen proinsulin to gold nanoparticle carriers effectively prevented diabetic onset in NOD mice and has been suggested for applications in islet cell graft tolerance.^[^ ^20^, ^136^ ^]^ Additionally, the targeted delivery of cyclosporine A using polylactide nanoparticles has also shown immunosuppressive effects, though this remains to be explored within the context of islet transplantation.^[^ ^137^ ^]^ Many tolerogenic approaches for local graft acceptance involve the use of immunoisolating barriers and are considered as cell encapsulation, which falls outside the scope of this open‐system review but is covered in a rich body of literature.^[^ ^13^, ^14^, ^18^, ^138^ ^]^

The use of biomaterials to enhance allograft acceptance has been explored through diverse techniques and methods, and again confers many benefits, such as allowing for a more localized and targeted administration of immunomodulating compounds—that might otherwise have problematic effects if given systemically—as well as the ability to deliver factors like cytokines or immunosuppressive in tandem. Biomaterial platforms have been demonstrated to release anti‐inflammatory compounds, including TGF‐*β*1 and dexamethasone, to aid in restoring normoglycemia following islet transplantation. Likewise, immunomodulatory agents such as IL‐33 and CTLA4‐Ig have been shown to upregulate helpful aspects of the immune system, and their localized delivery can enhance the durability of islet grafts. Lastly, factors that act as immunosuppressants, including Clodrosome and FK506, assist in creating a more favorable microenvironment to preserve transplanted islet viability.

Given that there are a multitude of tested ideas, the versatility of designing biomaterial strategies for islet allografts may prove useful for addressing disease heterogeneity and allow for the future combination of concepts presented here, such as a biomaterial platform both containing immobilized protein on its surface and loaded with anti‐inflammatory compounds for tightly regulated release. It is likely that a strong candidate providing a robust and long‐term restoration of normoglycemia with the accompanying islet graft will utilize an interdisciplinary and multifaceted approach. As our understanding of disease onset and the genetic and signaling pathways involved is enhanced, so will be the possibilities for immunomodulation through biomaterials and drug delivery. Many of the current methods have proven initial efficacy in animal models, but need to address limitations, such as depletion of the desirable administered compound over time and/or eventual immune graft infiltration via the foreign body response, before moving out of the preclinical phase. A focus on tolerogenic means, to allow for host immune system acceptance of allogeneic islets and *β* cells, will likely direct the future of this subfield. Again, these biomaterial approaches confer the benefit of providing local, targeted delivery of molecules and factors as opposed to the often times more harmful alternative of systemic immunosuppression, as well as offering a highly versatile platform for combinatorial strategies which can address multiple aspects of T1D and islet transplantation responses.

#### 2.2.3 Biomaterial Chemistry and Architecture

Beyond the use of biomaterials with the presentation or release of immune response mediators, altering material chemistry and architecture at the nano and microscales can also be employed as a strategy for immunomodulation (**Table** **2**). Though a biomaterial without biochemical cues cannot prevent allo‐rejection, it is important to consider the impact of material chemistry and topographical features on macrophage adhesion and activation during scaffold‐assisted islet transplantation, given that early interactions between the biomaterial and macrophages determine later activation and adhesion of lymphocytes.^[^ ^98^, ^139^ ^]^ Specifically, macrophage activation and immune response induction have been demonstrated to be significantly modulated by material size, geometry,^[^ ^140^, ^141^ ^]^ roughness,^[^ ^142^, ^143^ ^]^ and porosity.^[^ ^144^ ^]^ Additionally, there is interest in more passive methods of local immunomodulation which negate the need to incorporate bioactive mediators and design the materials such that delivery or presentation is controlled.

**Table 2:** Examples of biomaterial chemistry and architecture approaches to modulate immune responses

Biomaterial approach	Parameter	Example	Reference
Material properties	Chemistry	Chitosan HA PLGA PEG PDLLCL Methacrylic acid based	^[^^145^, ^146^^]^ ^[^ ^147^ ^]^ ^[^^148^, ^149^^]^ ^[^^150^, ^151^^]^ ^[^ ^152^ ^]^ ^[^^153^, ^154^, ^155^, ^156^^]^
	Surface property	Hydrophobicity	^[^^157^, ^158^, ^159^, ^160^, ^163^^]^
	Bulk property	Elasticity Molecular weight	^[^ ^164^ ^]^ ^[^^165^, ^166^^]^
Material architecture	Topography	Roughness Object coating Physical pattern	^[^ ^167^ ^]^ ^[^ ^142^ ^]^ ^[^ ^168^ ^]^
	Global features	Geometry Porosity Size Organization	^[^^140^, ^141^^]^ ^[^^169^, ^170^^]^ ^[^^171^, ^172^, ^173^^]^ ^[^ ^175^ ^]^

Materials such as chitosan,^[^ ^145^, ^146^ ^]^ hyaluronic acid (HA),^[^ ^147^ ^]^ PLGA,^[^ ^148^, ^149^ ^]^ PEG,^[^ ^150^, ^151^ ^]^ poly (d,l‐lactide‐*co*‐*ε*‐caprolactone) (PDLLCL),^[^ ^152^ ^]^ and methacrylic acid‐based materials^[^ ^153^, ^154^, ^155^, ^156^ ^]^ have been employed in the contexts of islet transplantation and local immunomodulation. For example, Smink et al., constructed a microporous scaffold consisting of PDLLCL and demonstrated a low inflammation score when compared to similar scaffolds.^[^ ^152^ ^]^ Material hydrophobicity is another design parameter that can be used. Akilbekova et al., demonstrated a nearly linear relationship between inflammatory cytokine levels and the surface hydrophobicity of latex beads.^[^ ^157^ ^]^ Given that the degree to which cells adhere to material surfaces is determined largely by the initial adsorption of protein and material hydrophobicity, non‐fouling materials with zwitterionic character may help create material surface more amenable to local immunotolerance.^[^ ^158^, ^159^, ^160^, ^161^, ^162^ ^]^ For example, Ladd et al., designed a non‐fouling, zwitterionic poly (carboxybetaine methacrylate) (PCBMA) polymer that prevented protein adsorption in serum and undiluted blood plasma.^[^ ^163^ ^]^

Other material‐specific characteristics, such as elasticity, may regulate foreign body responses. Irwin et al., demonstrated that fewer macrophages preferentially adhere to surfaces of lower moduli materials, while those with very high moduli minimized proinflammatory cytokine levels.^[^ ^164^ ^]^ Except elasticity, the specific molecular weight of the chains used can regulate immunomodulation. For example, larger HA chains have been demonstrated to confer local anti‐inflammatory capacities, while smaller chains may contrastingly serve as proinflammatory mediators.^[^ ^165^, ^166^ ^]^

Rather than manipulating the bulk material property, surface features can also be designed to modulate the immune response. Refai et al., demonstrated an increased secretion of proinflammatory cytokines such as TNF*α* by macrophages adhered to rough surfaces when compared to smoother polymer surfaces.^[^ ^167^ ^]^ Besides roughness, more intricate, heterogeneous designs across the surface architecture may more specifically control the immune response. These include designing, orienting, and aligning different topographical cues and structures. Zaveri et al., fabricated surfaces decorated with zinc oxide nanorods and demonstrated the adherence of significantly fewer macrophages when compared to flat surfaces.^[^ ^142^ ^]^ Chen et al., modified the existing material surface of different polymers directly via parallel gratings at the nano‐ and microscales and induced low inflammatory cytokine levels.^[^ ^168^ ^]^

Besides topography, global features of the biomaterial are important in modulating macrophage phenotype. Champion et al., demonstrated that the shape and geometry at the first point of contact with macrophages more strongly modulates their activity than size.^[^ ^141^ ^]^ Material porosity has also been demonstrated to play a role in the degree to which macrophages are activated and polarized.^[^ ^144^, ^169^, ^170^ ^]^ Also, studies have shown the role of microparticle size in determining the extent of the foreign body response.^[^ ^171^, ^172^, ^173^ ^]^ For scaffolds composed of multiple structures, the fiber size can be designed for immunomodulation. Wang et al., demonstrated that polyurethane (PU) nanofibers caused minimal macrophage responses in vitro and in vivo and induced only a mild foreign body reaction compared to larger PU microfiber membranes.^[^ ^174^ ^]^ In addition to pore size and fiber size, the way the fibers overlap and align can also determine the response.^[^ ^175^ ^]^

While many of these methods have yet to be implemented in islet transplantation and delivery, they nevertheless represent promising modalities that can offer a degree of passive modulation to foreign body reaction (FBR) in vivo. However, they are not without their challenges. An issue specific to designing physical and material features as manipulators of cell and immune responses is that these methods are largely static and fixed. Compared to the earlier‐discussed biochemical cues presented on surfaces or released by materials, systems relying on topographical cues to control cellular events are much more difficult to append with on‐demand stimulation or instruction. Moreover, mechanisms of how biomaterials with special chemistry and architecture affect the FBR are generally poorly characterized. As more advanced methods are developed and implemented to study the role of material surfaces in modulating foreign body responses, such as proteomics,^[^ ^176^, ^177^ ^]^ the impacts of surface chemistry and architecture are likely to be better characterized and elucidated.

### 2.3 Cell Co‐transplantation Strategy

**Figure 6:**
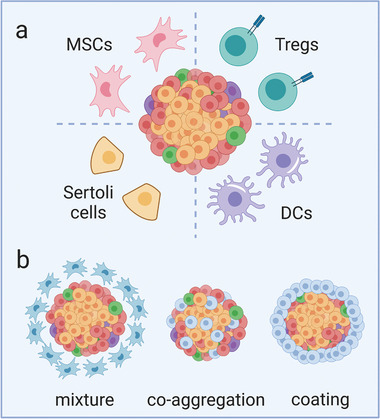
Cell co‐transplantation strategies. a) Types of immunomodulatory cells co‐transplanted with islets including mesenchymal stem/stromal cells (MSCs), regulatory T cells (Tregs), dendritic cells (DCs), and Sertoli cells. b) Co‐transplantation strategies with immunomodulatory cells: mixture, co‐aggregation, and coating.

Besides direct modification of islets and SC‐*β* cells and engineering biomaterials to present (deliver) ligands (drugs), the co‐transplantation of allogeneic islets with immunomodulatory cells—cells that are specifically able to deliver immunomodulatory signals to modulate local immune responses—has been investigated to delay allo‐rejection in *β* cell replacement. Recent research interests are focused on immunomodulatory cells including MSCs, Tregs, DCs, and Sertoli cells (**Figure** **6**a). Co‐transplantation of these cells with islets is typically based on one of three strategies: mixture, co‐aggregation, and coating (Figure 6b).

#### 2.3.1 MSC

MSCs are multipotent stem/stromal cells widely used in clinical applications aimed at tissue regeneration^[^ ^178^ ^]^ and immunomodulation.^[^ ^179^, ^180^, ^181^, ^182^, ^183^, ^184^ ^]^ MSCs have been demonstrated to inhibit allogeneic T cell responses through the secretion of regulatory cytokines/molecules such as transforming growth factor beta (TGF‐*β*),^[^ ^185^, ^186^ ^]^ IL‐10, nitric oxide (NO),^[^ ^187^ ^]^ indoleamine 2,3‐dioxygenase (IDO),^[^ ^188^, ^189^ ^]^ tumor necrosis factor‐inducible gene 6 protein (TSG6),^[^ ^190^ ^]^ IL‐1Ra,^[^ ^191^ ^]^ and prostaglandin E2 (PGE2). They are also responsible for the production of chemokines/chemokine receptors such as chemokine (C─C motif) ligand 2 (CCL2),^[^ ^192^ ^]^ and C─C chemokine receptor type 5 (CCR5) to recruit T cells into close proximity and downregulation of proinflammatory cytokines such as IFN*γ*,^[^ ^193^ ^]^ TNF*α*, and IL‐1*β*. Studies also showed that MSCs could promote generation of Tregs,^[^ ^194^ ^]^ induce DCs into a tolerogenic phenotype,^[^ ^195^, ^196^ ^]^ transform macrophages from a proinflammatory M1 to an anti‐inflammatory M2 phenotype,^[^ ^197^, ^198^ ^]^ and inhibit the proliferation of NK cells.^[^ ^199^, ^200^ ^]^

Given the immunoregulatory property of MSCs, many researchers have co‐transplanted MSCs with allogeneic islets and shown that MSCs suppressed T cell activity and improved graft survival.^[^ ^201^ ^]^ Specifically, Ding et al., reported that BALB/c mouse MSCs co‐transplanted with allogeneic C57BL/6 mouse islets under the kidney capsule of immunocompetent BALB/c mice prolonged islet grafts from 30 days (untreated) to 95 days (MSCs treated) by secreting matrix metalloproteinases (MMPs), MMP‐2, and MMP‐9, to reduce expression of CD25 on responding T cells.^[^ ^202^ ^]^ Li et al., showed that MSCs reduced the Th1/Th2 ratio, number of naïve T cells and memory T cells, suppressed DC maturation and secretion of IL‐12, and prolonged normoglycemia from 16 days (untreated) to 30 days (MSCs treated), when MSCs were co‐transplanted with allogeneic BALB/c mouse islets under the kidney capsules of diabetic C57BL/6 mice.^[^ ^203^ ^]^ Westenfelder et al., demonstrated that intraperitoneal administration of neo‐islets generated by co‐aggregation of allogeneic C57BL/6 MSCs and islet cells in NOD mice can prevent auto‐ and allo‐rejection for 77 days by promoting expansion of Tregs and upregulation of TGF‐*β* and IDO.^[^ ^204^ ^]^ Kim et al., reported that the combination of autologous MSCs and subtherapeutic dose cyclosporin A (CsA) further extended islet survival to 100 days after allogeneic rat islet transplantation through increased secretion of IL‐10 by CD11b^+^ cells and population of IL‐10 induced Tregs.^[^ ^205^ ^]^ Berman et al., showed that allogeneic monkey MSCs significantly promoted intraportal islet engraftment and function at 1 month posttransplant, compared with monkeys that received islets without MSCs. They also found that stable islet allograft function was associated with increased numbers of Tregs in peripheral blood.^[^ ^206^ ^]^

Despite the fact that co‐transplanting MSCs can delay allo‐rejection shown by studies above, long‐term engraftment (more than 100 days) is not yet reported. Though the function of MSCs has been demonstrated in multiple studies, the survival and engraftment of MSCs itself will heavily influence the longevity of immune protection over allogeneic islets. In addition, autologous MSCs potentially need to be obtained by getting patients’ tissues, digesting into single cells and sorting with specific markers, which can be time‐consuming, and the function of these cells are not guaranteed.

#### 2.3.2 Tregs

Tregs are a unique subset of immune cells which are characterized by CD4^+^CD25^+^FOXP3^+^ markers. Tregs play an important role in maintaining homeostasis and tolerance in the immune system. Tregs can suppress the activation, proliferation, maturation, and effector functions of a wide range of immune cells in vitro and in vivo, including CD4^+^ and CD8^+^ T cells, NK cells, B cells, and antigen‐presenting cells such as DCs, by secreting immunosuppressive factors (e.g., IL‐10 and TGF‐*β*) and interacting with cells through cell–cell contacts.^[^ ^207^, ^208^ ^]^ The therapeutic potential of Tregs has been demonstrated in preclinical trials to treat T1D.^[^ ^209^, ^210^, ^211^ ^]^

Specifically, Luo et al., investigated the efficacy of Tregs in blocking autoimmune destruction of syngeneic islet grafts in NOD mice. Tregs and islets were co‐transplanted in the kidney capsule of diabetic NOD mice. They observed a significant prolongation of graft survival by applying Tregs.^[^ ^212^ ^]^ Graham et al., reported long‐term graft protection by co‐delivery of islets and Tregs on microporous PLG scaffolds implanted on the intra‐abdominal fat in NOD mice.^[^ ^213^ ^]^ The euglycemia, which indicated delayed rejection, was maintained as long as 100 days with Treg co‐transplantation. However, for Tregs delivered systemically, no protection was observed. This study demonstrated that co‐localization of Tregs with islets could protect islets from destruction. To understand the role of Tregs in allotransplant tolerance, Naohiro et al., co‐aggregated Tregs from C57BL/6 mice and islets from BALB/c mice and transplanted them in the liver of diabetic C57BL/6 mice without systemic immunosuppression. They found that Tregs randomly distributed in islet aggregates enabled long‐term survival of allogeneic islet grafts in the liver for more than 100 days.^[^ ^214^ ^]^

Unlike systemic infusions of autologous Tregs to treat T1D, which have been extensively carried out and even advanced to phase 1 clinical trials,^[^ ^207^, ^208^, ^209^, ^210^, ^211^ ^]^ co‐transplantation of Tregs with human islets is not well investigated. Regardless of Tregs administration pathway in patients, the mass production of Tregs and the maintenance of long‐term Treg function still need to be further investigated.

#### 2.3.3 Dendritic Cells

DCs are professional antigen presenting cells and play an important role in inducing and maintaining immune tolerance. DC immunotherapy can induce tolerance to specific alloantigens, which might be a better candidate over generalized immunosuppression. Targeting DCs through different DC surface molecules showed effective modulation of immune responses and prolonged transplant survival in islet transplantation.^[^ ^215^ ^]^ Studies have shown that tolerogenic DCs favored allo‐graft acceptance in islet transplantation through systemic injection. Researchers have reported using these types of tolerogenic DCs in their studies: host immature DCs,^[^ ^216^ ^]^ in vivo allopeptide‐primed DCs,^[^ ^217^ ^]^ drug treated mature autologous DCs^[^ ^218^ ^]^ and gene (IL‐10, CTLA4‐Ig, and GAD65/DCR3) modified DCs.^[^ ^219^, ^220^, ^221^ ^]^ Huang et al., demonstrated that donor (BALB/c) but not recipient (C57BL/6) DCs, pretreated with recipient kidney‐derived MSCs, significantly delayed graft rejection (*P* < 0.01), when donor DCs were co‐transplanted with donor islets into the kidney capsule of diabetic C57BL/6 mice. MSCs significantly inhibited MHC class II expression, while increasing CD80 expression on DCs in both syngeneic and allogeneic co‐cultures. They reasoned that the delay was caused by inhibiting alloreactive CD4^+^ T cell proliferation, IgM^+^/CD19^+^ mature B cell generation and antibody (IgG and IgM) production. Interestingly, co‐cultured recipient DCs failed to promote graft survival, which might be related to the strength of the direct allo‐recognition being activated in the early stage after transplantation.^[^ ^196^ ^]^ Similar to Tregs, the generation and maintenance of tolerogenic DCs still remains an issue.

#### 2.3.4 Sertoli Cells

Sertoli cells play a crucial role in creating the immune‐privileged environment of the testis by forming tight junctions that make up the blood‐testis barrier, which prevents the passage of lymphocytes and antibodies.^[^ ^222^ ^]^ Sertoli cells secrete immune‐modulating factors, such as TGF‐*β* ^[^ ^223^ ^]^ and clusterin.^[^ ^224^ ^]^ Sertoli cells also express Fas ligand, which can eliminate Fas‐positive activated T lymphocytes.^[^ ^225^, ^226^ ^]^ Korbutt et al., reported that islet graft survival could be improved by co‐transplantation of the islets with Sertoli cells under the kidney capsule in a rat allotransplantation model.^[^ ^227^ ^]^ Takeda et al., showed that transplanting allogeneic C3H islets under the kidney capsule of diabetic C57BL/6 mice together with C3H testicular tissue significantly prolonged the survival of islet allograft.^[^ ^228^ ^]^ They reasoned that Fas ligand highly expressed in testicular tissues were responsible for protection from allograft rejection by inducing apoptosis of anti‐graft activated T cells. Takemoto et al., demonstrated in their study that transplanting co‐aggregates of islets cells and Sertoli cells from BALB/c mice into C57BL/6 mice via the portal vein resulted in long‐term normoglycemia for more than 100 days.^[^ ^229^ ^]^ Moreover, Rafael et al., co‐transplanted neonatal porcine islets with porcine Sertoli cells inside a subcutaneous collagen‐covered device in T1D patients without immunosuppression. They showed that half of the patients reduced their exogenous insulin use for up to 4 years. Two patients became insulin independent for several months.^[^ ^230^ ^]^ These results were promising; however, no significant follow‐up studies were reported.

#### 2.3.5 Other Types of Cells

There are also other types of cells that have been explored in co‐transplantation with islets.^[^ ^231^, ^232^ ^]^ For example, Jalili et al., engineered syngeneic fibroblasts using adenovirus to overexpress IDO, which is a tryptophan degrading enzyme and helps maintain maternal tolerance toward the fetus. The IDO‐expressing fibroblasts were co‐transplanted with allogeneic islets within collagen gel matrix in the kidney capsule of diabetic immunocompetent mice. They found that IDO expressing composite allograft survived and functioned significantly longer than controls (41.2 ± 1.64 vs. 12.9 ± 0.73 days). IDO expressing cells not only evidently prevented lymphocyte infiltration but also delayed the production of donor specific alloantibodies.^[^ ^233^ ^]^

Compared to systematic injections, co‐transplantation of immunomodulatory cells with islets can generate a local immunotolerant microenvironment. The feasibility of translation is however limited by several factors: large amount of autologous immunomodulatory cells needed, survival and engraftment of these cells, and maintenance of long‐term cellular function.

## 3 Conclusion, Challenges, and Outlook

The successful application of islet transplantation in some T1D patients has demonstrated the potential of cell therapy as a therapeutic option to restore physiological glucose control. However, two major barriers, shortage of donor cells and side effects of systematic immunosuppression, have limited its wide use. With the advancement of stem cell technology that makes it possible to provide unlimited number of insulin‐producing cells,^[^ ^63^, ^76^, ^234^, ^235^, ^236^, ^237^ ^]^ avoiding or mitigating the use of systematic immunosuppression has become a research area of key interest. Cell encapsulation or immunoisolation is one way to tackle this problem, but it has its own challenges including inability to directly integrate with the host and the FBR causes a decline in cell viability in the long‐term.^[^ ^13^, ^18^, ^76^, ^238^, ^239^, ^240^, ^241^, ^242^ ^]^ In this review, we discussed the immune responses against nonencapsulated islet cells and various ways to induce local immunosuppressive or immune tolerant environment to overcome rejection and improve graft survival. Although progress has been promising, many hurdles remain.

Transplanting solely hypoimmunogenic primary islets or insulin‐producing *β* cells seem to be very straightforward because it does not require third‐party biomaterials or supporting cells. It is noted that surface or genetic modifications are cumbersome to primary islets and could compromise the viability and function of islets. While modifying human islets seems unrealistic in clinical applications, engineering universal cells from pluripotent stem cells that can evade immune recognition holds great potential in diabetes cell therapies. Despite these advances, crucial questions remain such as the retention of genomic and epigenetic changes over time and the stability of cell phenotypes. Furthermore, the safety issues of these hypoimmunogenic cells also should be well managed because cells that evade immunity system are inherently risky. Similarly, there is a possibility of undifferentiated stem cells which may become a teratoma in vivo. Even though some groups developed safe‐cell system by integrating “suicide gene” into stem cells to ensure the safety,^[^ ^243^ ^]^ it is still unclear how these cells behave in humans long‐term and may require further investigation.

In other efforts, biomaterials coupled with immunomodulation provide multifaceted tools to locally modulate immune responses and represent an interesting approach to aid cell transplantation. There are clear advantages in this strategy including the safety as “nonliving” materials and readiness as an “off‐the‐shelf” product. Biomaterials are relatively easy to be prepared in mass‐production, while modification on the *β* cells or preparing immunomodulatory cells are often complicated in addition to the requirement of good manufacturing practices that must meet clinical standards. However, the long‐term durability of biomaterial and delivery approaches remains challenging given the limited presentation of immunomodulatory ligands and eventual depletion of loaded functional reagents. Thus, retention of the agents delivered or a system that allows refilling of these proteins and molecules needs to be developed in the future.

Immunomodulatory cells act as “living” drug reservoirs and once engrafted may increase functional durability, through continuous production of cytokines or expression of surface markers to modulate the immune system. However, problems such as the localization of these cells with *β* cells, maintenance of their immunoregulatory function and longevity of the cells are all need to be considered. In addition, the acquisition of these immunomodulatory cells is not easy given the fact that they are either autologous or rare in cell population. Thus, improvements in the acquisition, retention, stability, potency, and localization of these immunoregulatory cells are needed to improve their safety and efficacy.

As we progress in developing treatments and cure for T1D, a functional solution will likely require a holistic approach making use of multiple immuno‐modalities and tissue engineering approaches. It is possible in the future that all three approaches—an engineered tissue construct consisting of immuno‐invisible *β* cells and immunoregulatory accessary cells within 3D immunomodulatory biomaterials—could be combined to create long‐term functional and safe cell therapies for T1D. It is also important to consider the heterogeneity of the disease and tailor the therapeutic approaches to achieve optimal outcome.^[^ ^16^, ^244^ ^]^ Finally, although not discussed in this review, vascular and nervous systems must be taken into account in order to develop a truly functional cure for this disease.^[^ ^245^, ^246^, ^247^ ^]^

## Conflict of Interest

The authors declare no conflict of interest.
